# Coordinated decision-making boosts altruistic motivation—But not trust

**DOI:** 10.1371/journal.pone.0272453

**Published:** 2022-10-04

**Authors:** Matthew Chennells, Mateusz Woźniak, Stephen Butterfill, John Michael

**Affiliations:** 1 Department of Philosophy, University of Warwick, Coventry, United Kingdom; 2 Department of Cognitive Science, Central European University, Vienna, Austria; Texas A&M University, UNITED STATES

## Abstract

In the current study, we separately tested whether coordinated decision-making increases altruism and whether it increases trust. To this end, we implemented a paradigm in which participants repeatedly perform a coordinated decision-making task either with the same partner on every trial, or with a different partner on each trial. When both players coordinate on the same option, both are rewarded. In Experiment 1 (*N =* 52), participants were sometimes presented with tempting opportunities to defect. In Experiment 2 (*N =* 97), participants sometimes had to decide whether or not to trust that their partners had resisted such tempting opportunities. The results show that repeatedly coordinating with the same partner increased participants’ resistance to temptation (Experiment 1) but did not increase trust (Experiment 2). These findings support the hypothesis that coordinating with a partner increases altruistic motivation towards that partner; they do not support the hypothesis that coordinating boosts trust.

## Introduction

The versatility and flexibility of human cooperation is unparalleled by any other species. We routinely work together to achieve ends that we could not achieve alone, even setting aside short-term interests to maximize the benefits to our interaction partners and larger social groups. In recent decades, a substantial body of research in evolutionary theory, experimental economics and psychology has been devoted to investigating the evolutionary origins of human cooperation [1–5].

This research on the evolution of cooperation also informs and constrains research into the cognitive and motivational mechanisms that *proximally* support cooperation. For example, theoretical work on the evolution of cooperation–specifically, on direct [6] and indirect reciprocity [7], competitive altruism [8], and the interdependence hypothesis [8]–provides us with reasons to expect that people’s willingness to cooperate with another agent should be strengthened by any cue that one is likely to interact with that agent in the future. One such cue could be the act of coordination itself: when two agents have coordinated with each other–i.e. when they mutually adapt their decisions or actions to bring about a shared goal or compatible but distinct individual goals [4, 9]–each may come to perceive the other as an in-group member or a valuable partner, or both. If so, then coordination with a partner now may boost people’s willingness to cooperate with that partner in future–i.e., to coordinate with them despite the availability of alternative options which may be individually preferable [9]. And indeed, previous research has shown that people’s willingness to cooperate in social dilemmas may be fostered by repeated coordination in decision-making [10, 11] or in action [12, 13].

This raises the further question as to what the specific cognitive and motivational mechanisms are by which coordination increases cooperation. One possibility is that *coordination may enhance trust in one’s partner*. This is the hypothesis put forward by Rusch and Luetge [11]; we will refer to it as the ‘trust hypothesis’. They reasoned that coordination with a partner may lead people to perceive their partner as being reliable in general, and therefore also as someone who is likely to resist the temptation to behave selfishly. As a result, people should be more likely to cooperate with a partner with whom they share a history of coordination. And indeed, this rationale is consistent with the results of an earlier study [14] in which it was shown that cooperation rates in a prisoners’ dilemma were higher if participants had previously performed a coordination game together than if they had not. Building on this, it was found [11] that cooperation rates in a prisoners’ dilemma were boosted when rounds of the prisoners’ dilemma were interspersed among rounds of a coordination game (i.e. the stag hunt) played together with a fixed partner.

But while the trust hypothesis may explain why people would be more likely to expect their partners to cooperate in a prisoners’ dilemma when they have repeatedly been coordinating with the same partner than when they have been coordinating with different partners, it does not directly explain why people would then themselves be motivated to cooperate. Indeed, if one expects the other player in a prisoners’ dilemma to cooperate, then one can expect to attain the highest possible reward by defecting. An increase in trust could only explain why people who want to cooperate do not defect in order to avoid being exploited, not why they would be willing to cooperate at a cost to themselves. Moreover, it is worth noting that a track record involving feedback on the results of coordination with a partner who coordinated when it was in her interest to do so does not directly provide evidence that that partner would resist tempting alternatives if they were to arise. Taken together, these considerations provide grounds to scan the conceptual landscape for potential alternative hypotheses to explain the documented effects of coordination upon cooperation in prisoners’ dilemmas.

Our starting point in this regard is the observation, alluded to above, that cooperation in prisoners’ dilemmas requires not only trust that the other player will cooperate, but also a willingness to pay a cost in order to cooperate. On this basis, the alternative hypothesis that suggests itself is that *coordination may increase cooperation by eliciting altruistic motivation*–i.e. by boosting the willingness to pay a cost in order to cooperate. In referring to the willingness to pay a cost to cooperate as an altruistic motivation, we are relying on ‘a behavioural—in contrast to a psychological—definition of altruism as being [willing to perform] costly acts that confer economic benefits on other individuals’ [15, p.1]. In this sense, an altruistic motivation is any motivation to perform costly acts that benefit others, and the hypothesis under consideration, which we will call the ‘altruism hypothesis’, is that coordination boosts any motivation to perform costly acts that benefit others.

Accordingly, the altruism hypothesis that we are considering here is broad in the sense that it is consistent with a range of specific hypotheses about the nature of the altruistic motivation elicited by coordination (i.e. the proximal mechanism), and also with a range of specific hypotheses about evolutionary origins (i.e. the distal mechanism). For example, it is consistent with a line of reasoning based upon the interdependence hypothesis, which states that human cooperation arose in a period in which our ancestors lived in small groups of individuals whose interests were largely interdependent, and for whom it was therefore not typically beneficial to act selfishly to the detriment of other group members [8]. Insofar as repeated coordination with a partner may provide a cue that one has a stake in their welfare, this line of reasoning suggests that coordination may boost cooperation by boosting altruistic motivation towards one’s partner. But the altruism hypothesis is also consistent with direct or indirect reciprocity, as well as competitive altruism, insofar as repeated coordination may indicate that one is likely to interact with the same partner in the future or that the interaction is relevant for one’s reputation.

Moreover, the altruism hypothesis under consideration here is also consistent with the idea that a history of collaboration with a partner creates a sense of debt, boosting altruistic sharing with that partner due to feeling obligated to share or be generous [16]. The altruism hypothesis is also consistent with the idea that coordination makes social expectations salient and thereby gives rise to a sense of commitment to one’s coordination partner [17, 18]. And it is consistent with the conjecture that interpersonal synchrony and minimal forms of interpersonal engagement trigger altruistic behaviour [19–23].

Research to date has not distinguished the trust hypothesis from the altruism hypothesis. For example, the authors of one study conclude simple reciprocal interactions lead children to believe their relationships are characterised by *mutual* care and commitment [21]. Yet their evidence of children acting being more willing to act for others’ benefit after coordinating is consistent with the altruism hypothesis under consideration: the additional commitment that coordination leads to a level of *mutual* care for each other is not further tested. Importantly, a similar point applies to the studies using prisoners’ dilemmas to measure agents’ willingness to cooperate [11, 14] which are used to support the trust hypothesis: as altruistic motivation can increase participants’ willingness to cooperate even in the absence of trust, it is difficult to determine to what extent, if at all, people’s trust that their partner will cooperate mediates cooperation rates in prisoners’ dilemmas.

### The current research

**Fig 1:**
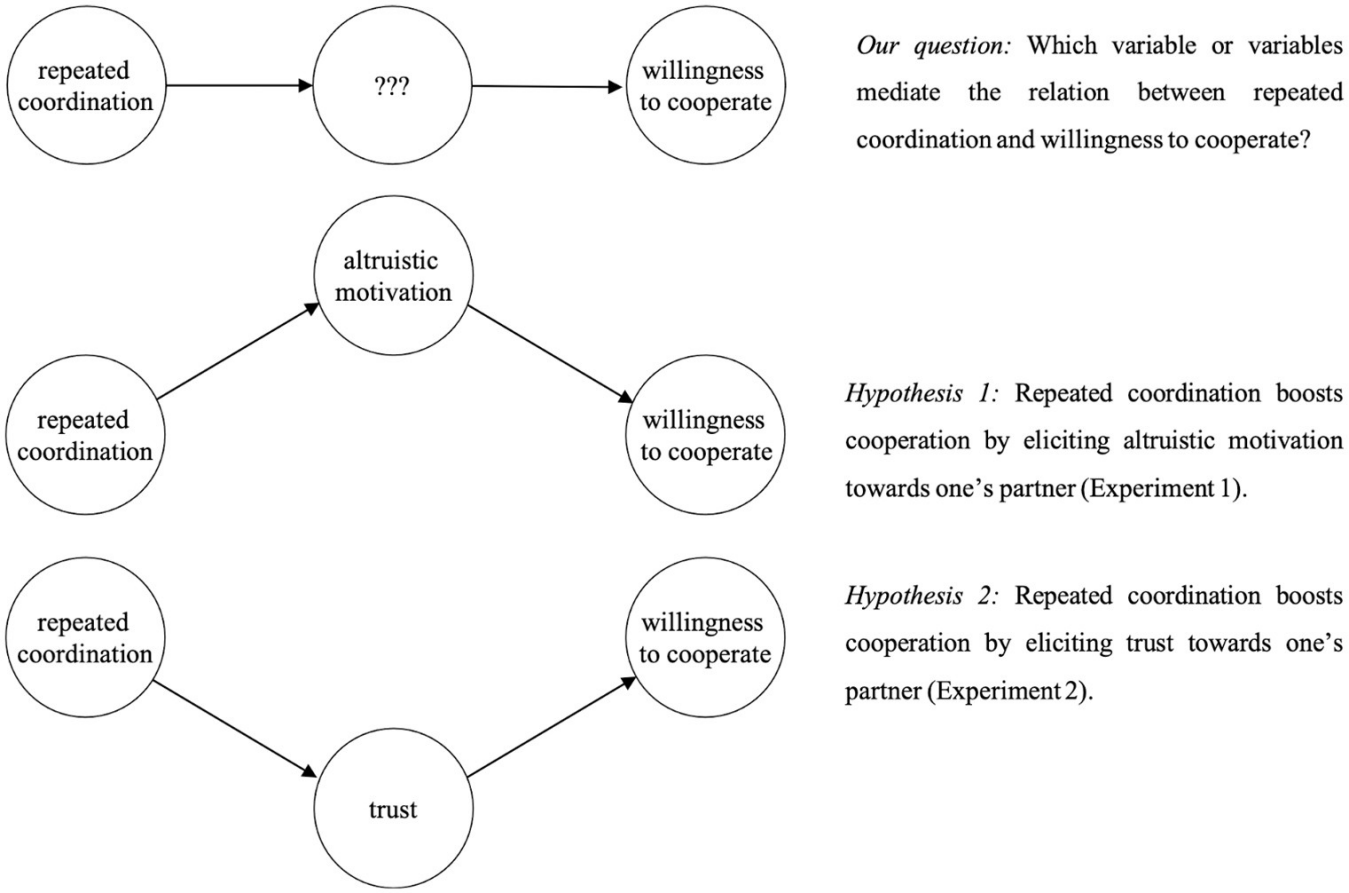
The current study aims to answer the question of why it is that repeated coordination may boost one’s willingness to cooperate with one’s partner, by testing separately for the emergence of altruistic motivation (Hypothesis 1) and trust (Hypothesis 2) towards one’s partner.

We conducted two pre-registered experiments. As outlined in Fig 1, the experiments separately tested two distinct, albeit compatible, hypotheses about the mechanisms by which coordination increases cooperation. We aimed in Experiment 1 to investigate whether coordination might boost cooperation via an increase in altruistic motivation (the altruism hypothesis), and in Experiment 2 to investigate whether it does so via an increase in trust (the trust hypothesis). The experiments were designed to tease apart the potential effects of altruistic motivation and trust, with each study design isolating one of these hypotheses. Importantly, our main interest was in the effect of coordination *per se*; that is, whether the mere act of coordinating with a partner cued one or both proximal mechanisms, independently of evidence participants receive about their partners’ behaviour (i.e. feedback). Our two hypotheses were as follows:

*The Altruism Hypothesis (Hypothesis 1)*: Repeated coordination elicits altruistic motivation towards one’s partner (Experiment 1).
*The Trust Hypothesis (Hypothesis 2)*: Repeated coordination elicits trust towards one’s partner (Experiment 2).

## Experiment 1

To test Hypothesis 1, Experiment 1 probed the effects of repeated coordination upon altruism *independently of trust*. To this end, we implemented a sequential joint decision-making task in which participants could choose whether or not to coordinate with a partner. We varied whether and to what degree the option not to coordinate constituted a temptation and measured the frequency with which participants chose to coordinate despite this temptation (altruism rates). In a within-subjects design, we manipulated the partner’s relationship: in one experimental block, participants played with the same partner on every trial (Fixed Partner Condition), whereas in a separate experimental block they played with different partner on each trial (Variable Partners Condition). Crucially, the choices made by their partners could not affect them negatively, and they were informed that their partners would receive no feedback about their choices. This ensured that participants’ willingness to coordinate could only be explained by altruistic motivation, not by trust or by any expectation of reciprocity.

The data from both experiments, as well as the base regression models mentioned in Tables 1 and 3, can be found at: https://osf.io/f4rqj/. The pre-registration for this experiment can be accessed at: https://osf.io/fnj6r/.

**Table 1:** Analysis results from Experiment 1 using mixed-effects logistic regressions of partner condition and temptation level on subject’s choices. Model 1 is used for inferring the effect of partner condition on altruism rates; it contains a by-subject random coefficient for this variable. Likewise, Model 2 is used for inferring the effect of temptation level on altruism rates; it contains a by-subject random coefficient for this variable.

Dependent variable = Altruism choice	Model 1					Model 2				
			95% CI for odds ratio					95% CI for odds ratio		
	B (SE)	*p* =	Lower	Odds Ratio	Upper	B (SE)	*p* =	Lower	Odds Ratio	Upper
Partner Condition	0.426	.038	1.023	1.532	2.371	0.301	.029	1.025	1.351	1.782
	(0.205)					(0.138)				
Temptation Level	-0.898	.000	0.390	0.407	0.425	-1.277	.000	0.190	0.279	0.397
	(0.022)					(0.181)				
Constant	1.162	.001	1.594	3.195	6.546	0.252	.582	0.510	1.288	3.255
	(0.352)					(0.460)				
By-subject random coefficient	Partner Condition					Temptation Level				
Observations	8,320					8,320				
Log Likelihood	-2,813.80					-2,394.80				
AIC	5,641.60					4,803.70				

Dependent variable is a choice dummy equal to 1 if subject chose altruistic option and 0 if alternative option. Partner Condition dummy equal to 1 in Fixed Partner Condition and 0 in Variable Partners Condition. Both regressions include as covariates a by-subject random intercept and a by-subject random coefficient for trial number.

### Participants

Using G*Power 3.1 [24] we determined that a sample size of 52 in a within-subjects design would provide 80% statistical power for detecting a medium-sized effect (Cohen’s d = 0.4) equivalent to what we observed in a pilot study. We therefore recruited 52 participants (33 females, 18 males, 1 other; age range: 18–40, *M* = 21.9, *SD* = 4.4). All participants were recruited through the University of Warwick SONA System. All participants reported speaking and understanding English. Participants provided their informed written consent prior to the testing. Ethics clearance for Experiment 1 was obtained from the University of Warwick Humanities and Social Sciences Research Ethics Committee (HSSREC), and all methods were performed in accordance with the Declaration of Helsinki.

### Apparatus and stimuli

The experiment was displayed on a 24-inch wide screen (16:9) computer monitor (resolution: 1920 x 1080 pixels, framerate = 60Hz.), consistent across participants and experiment sessions. The program for the experiment was written in Open Sesame [25]. The two choice options were presented as 7 cm x 5 cm rectangular fields, separated by 36 cm. The mouse start field was also 7 cm x 5 cm and was positioned 18 cm lower at the midpoint between the two choice options. During trials, participants used the mouse to select by clicking on one of their choice options. The computer screen provided participants with real-time visual feedback on their inputs.

### Design

Experiment 1 had a 2 (Partner Condition: Fixed Partner vs. Variable Partners) x 8 (participant reward temptation level) repeated measures design. Both factors were within-subject.

### Procedure

After participants had given their informed written consent, they were told that they would be paired with various partners during the experiment. They performed the experiment in group sessions of 12–16 people. Participants were instructed that both their own and their partner’s monetary payments at the end of the experiment would depend on the points accumulated during their task. They were informed that in addition to the £5 show-up fee, they would also be paid a bonus up to a maximum £5 based on the number of points they earned during one randomly selected trial of the experiment, and that the same was true for their partner(s). Instructions were displayed on the screen and participants read them on their own. Participants were encouraged to take as much time as they needed and had the opportunity to ask clarification questions during the instructions phase; questions and their answers were repeated in public for all participants to hear. The task began when all participants confirmed that they understood the instructions. At the beginning of the experiment participants were assigned a player number between 0 and 20. They were told that their partner(s) would see this number when interacting together, that they would, in turn, also see their partner’s number, and that these numbers remained constant throughout the experiment. Participants were thus led to believe they were interacting with real people; in reality, player numbers were pre-set and participants interacted with pre-programmed virtual partners.

The experiment lasted approximately 40 minutes, during which participants performed two experimental blocks, one with a Fixed Partner and one with Variable Partners. The order of blocks was counterbalanced across participants. In each experimental block participants underwent an induction phase consisting of 10 trials, which was immediately followed by a test phase consisting of 80 trials, making both phases look like one uninterrupted block.

**Fig 2:**
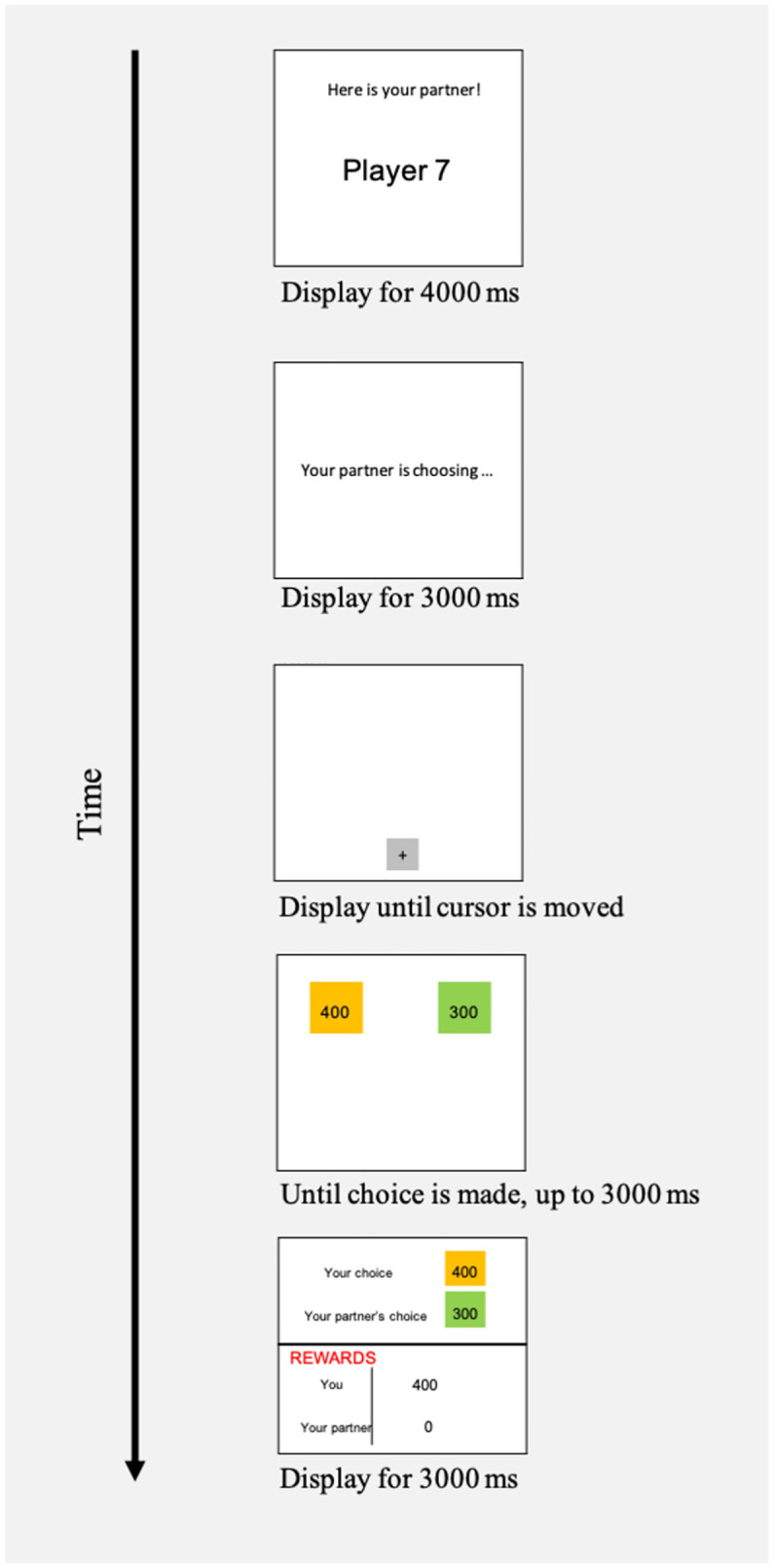
Experiment 1 trial structure. At the beginning of each trial, an image of the partner’s player number was displayed, which was either the same (Fixed Partner Condition) or different (Variable Partners Condition) on every trial. Then, the partner chose one of two values. The participant did not see what these two values were, and did not see what the partner had chosen, but was then herself presented with two values to choose between. One of these, indicated in green or blue, was the same value that the partner had chosen (coordination option); the other, indicated in orange, was an alternative value (alternative option). We varied whether, and to what extent, the alternative option constituted a temptation.

**Fig 3:**
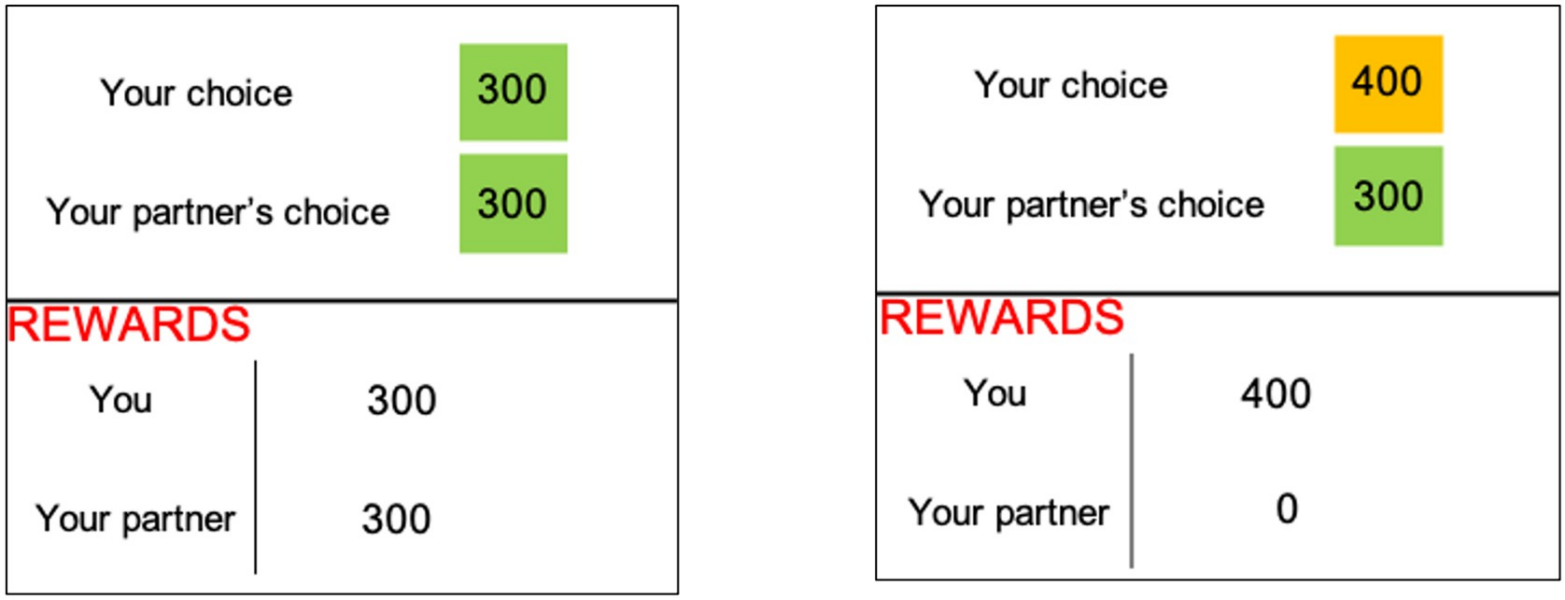
Experiment 1 payoff structure. Payoffs for each trial were determined as follows. If the participant chose the coordination option, then each received the corresponding amount (left box above). If the participant chose the alternative option, then the participant received the amount corresponding to the alternative option and their partner received no reward (right box above). Participants were thus aware that both their and their partner’s payoffs depended on the choice they made. Payoffs were displayed for 3000 ms before participants proceeded to the next trial. The amounts associated with the payoffs for participants’ coordination and alternative options varied, over pre-specified intervals and ranges, unpredictably across trials.

At the beginning of each trial (See Figs 2 and 3), a partner’s player number was displayed for 4000ms, which was either the same (Fixed Partner Condition) or different (Variable Partners Condition) on each trial within a block. Afterwards a message “Your partner is choosing…” was displayed for a fixed duration of 3000 ms, to indicate to participants that their partner was choosing between two unseen options. The time was kept constant between trials so that participants could not infer anything about their partner’s decisions from the timing. Then, the participant was presented with a blank screen including a grey square and fixation cross where the mouse cursor began. Moving the cursor out of this square revealed to the participant two values to choose between. One of these, indicated in green or blue, was the value that their partner had chosen (coordination option); the other, indicated in orange, was the alternative value (alternative option). If the participant chose the coordination option, then both the participant and the partner would each receive the amount of points corresponding to the selected value. If the participant chose the alternative option, they received that amount of points, while her partner did not receive any points. The alternative option was tempting when its value was greater than that of the coordination option, and the level of temptation which it presented was a function of the value of the alternative option. The values for the coordination options ranged from 250 to 450 points in increments of 50. The value of the alternative option was either 100 or 50 points lower, the same, or 50, 100, 150, 200, or 250 points higher than the coordination option, leading to eight different temptation levels present during the test phase within each block (resulting in 10 trials with each temptation level per block) presented in random order. In contrast to the test phase, the induction phase included 10 trials (presented in random order) with the following temptation levels: 4 trials in which the alternative option was 100 points less than the coordination option; 4 in which it was 50 less; and 2 in which it was the same. In each phase the position of the two options (coordinative and alternative) varied equally between the left- and right-hand sides of the screen, and the colour of the coordination option varied equally between blue and green.

A trial proceeded when participant mouse-clicked on one of two options or after 3000 ms elapsed. After the participant made a choice, there was a 500 ms delay, and the payoffs were displayed for 3000ms. The payoff display showed the participant’s choice, their partner’s ostensible choice, and the payoffs for each player. Participants were told that their partner would receive no feedback on the choices the participant made until one trial was randomly selected for payment at the end of the experiment. Payment was automatically calculated based on the choice made in the selected trial and participants were paid at the end of the experiment after all participants finished.

### Results

Hypothesis 1 predicts that altruism rates should be higher in the Fixed Partner Condition than in the Variable Partners Condition. To test this, we employed a mixed-effects logistic regression model on our dependent variable (DV), altruism rate, an indicator of participants’ trial-by-trial choice (0: alternative option; 1: coordination option). Our two independent variables (IVs) of interest in the model were partner condition, a dummy equal to 1 if the participant’s trial in question was in the Fixed Partner block and 0 if in the Variable Partners block, and temptation level, a numerical variable ranging from -2 to 5 indicating the attractiveness of the participant’s alternative option. We included a random effect to allow the intercept to vary by participant and a by-subject random coefficient for trial number to control for a time effect.

One concern when using multiple-regression models to analyse data for confirmatory hypothesis testing is the possibility of inflated Type I error rates when specifying models with random effects–i.e. when incorrect random-effect structures are specified in the model, which do not reflect random effects present in the underlying population or fail to control for random measurement error in the data [26, 27]. It has been shown [26] that this is of particular concern with data generated by experimental designs which involve within-subject manipulations and multiple observations per treatment level per unit and where fixed effects of interest are estimated and tested for significance, as in the case of our paradigm. Not including random coefficients for fixed variables in the sample risks giving overconfident estimates that fail to account for weaker conditional independence between multiple within-subject observations [28]. To control for this, we include in our analysis by-subject random coefficients for both of our fixed independent variables of interest. This allows for participants to differ in the slopes of their responses, thus accounting for the nonindependence of data points. Given the models’ failure to converge when both random coefficients are present, we follow [26] in pursuing separate analyses for each.

**Fig 4:**
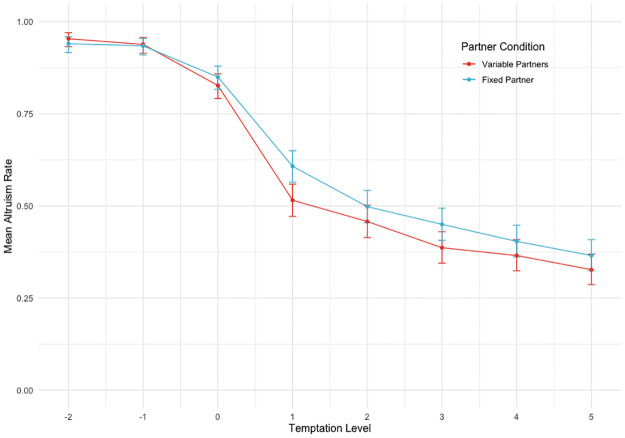
Experiment 1 data. Graphs show mean altruism rates (proportion of trials in which participants chose the coordination option) by Partner Condition for the corresponding temptation level (see: Methods) of participants’ alternative option. Points represent where empirical data was collected, and error bars represent 95% confidence intervals calculated using binomial tests. Regression results (see Table 1) show that altruism rates were significantly higher when participants coordinated with the same partner (Fixed Partner) on every trial than when coordinating with different partners (Variable Partners) on each trial. In addition, altruism rates are significantly decreasing with increases in the level of temptation of the alternative option.

Table 1 shows the results of two regression models, each including the by-subject random coefficient corresponding to one of the IVs–partner condition (Model 1) and temptation level (Model 2). Fig 4 presents the data from Experiment 1, showing mean altruism rates by partner condition and by temptation level.

Results shown in the column for Model 1 corroborate our prediction that altruism rates were significantly higher (*b* = 0.426, *p* = .038) when participants coordinated with the same partner (partner condition = 0) on every trial than when coordinating with different partners on each trial (partner condition = 1). The odds of subjects choosing the coordination option change by 1.53 (95% conf. int.: 1.02, 2.37) when they coordinate with a fixed partner relative to coordinating with variable partners.

As a manipulation check, we also predicted a negative main effect of temptation level (Model 2), with participants more likely to choose the alternative option as the payoff for doing so relative to the coordination option increased. The results confirm this prediction: temptation level is significantly associated with reduced altruism rates (*b* = -1.277, *p* = .000), all else constant. From Model 2, an increase in temptation level by one unit is associated with a 0.28 (95% conf. int.: 0.19, 0.40) change in the odds of an altruistic choice.

Finally, we ran an additional exploratory statistical test (not shown here; see Data Availability section), categorising temptation level into two factors (one factor for all levels less than or equal to 0 and another for all levels greater than zero; that is, temptation to act selfishly is either present or absent) and checking for an interaction with partner condition. We found a significant main effect of temptation level though not of partner condition, while finding an interaction between the two, implying that participants were more likely to cooperate with a fixed versus variable partner but only when there was a temptation to defect, thus supporting our hypothesis.

## Experiment 2

In Experiment 2, we adapted the paradigm used in Experiment 1 to investigate whether repeated coordination enhances trust (Hypothesis 2). While in Experiment 1, participants unilaterally chose whether to coordinate and thereby benefit or harm their partners, in Experiment 2 it was the partner who unilaterally chose whether to coordinate. This meant that participants in Experiment 2, who were not informed about which choice the partner had made, had to decide whether to trust that the partner had coordinated (see Table 2), or take a smaller, but safe, option which did not rely on the partner’s choice.

**Table 2:** Overview of differences in how participant and partner are impacted by each other’s choices in Experiments 1 and 2.

	Who chooses whether to coordinate	Partner impacted by Participant’s choice	Participant impacted by Partner’s choice
**Experiment 1 (altruism, not trust)**	Participant only	yes	no
**Experiment 2 (trust, not altruism)**	Partner only	no	yes

To this end, in Experiment 2 we introduced a new game that differed in two ways from the previous game. First, participants could choose whether or not to trust their partners, where choosing to trust would potentially gain participants a higher reward but also implied a risk of receiving a lower reward if the trust was misplaced. Second, we ensured that participants’ choices in Experiment 2 had no effect on their partner’s payoffs, thereby excluding the possibility that any altruistic motivation could influence their choices.

The pre-registration for this experiment can be accessed at: https://osf.io/kepj8.

### Participants

Using G*Power 3.1 [24] we determined that for an increased statistical power of 95% a sample size of 88 would detect a medium-sized effect (Cohen’s d = 0.4) equivalent to what we observed in our previous study. Sessions were structured to include between 16–20 participants and our stopping rule was such that we included data from all participants up to and including those participating in the final session in which we crossed the participant threshold. We therefore recruited 97 participants (53 females, 44 males; age range: 18–35, *M* = 20.8, *SD* = 2.4), seated in the same computer lab under identical conditions as in Experiment 1. Participants who participated in Experiment 1 were not permitted to participate in Experiment 2. All participants were recruited through the University of Warwick SONA System. All participants reported speaking and understanding English. Participants provided their informed written consent prior to the testing. As with Experiment 1, ethics clearance for Experiment 2 was obtained from the University of Warwick Humanities and Social Sciences Research Ethics Committee (HSSREC), and all methods were performed in accordance with the Declaration of Helsinki.

### Apparatus and stimuli

The experiment was conducted using the same computers and settings (size, resolution, keyboard and mouse input) as in Experiment 1. The program for the experiment was written in JavaScript using the jsPsych toolbox [29]. Boxes were presented in a horizontal line on the screen, with partners’ choices separated from participants by a dark vertical line, while choice buttons were shown on a line below the boxes. During trials, participants responded using a computer mouse by clicking on one of two choice options. The computer screen displayed real-time visual feedback.

### Design

Experiment 2 had a 2 (partner condition: Fixed Partner vs. variable partner) x 8 (partner reward temptation condition) repeated-measures design. Both factors were within-subject.

### Procedure

**Fig 5:**
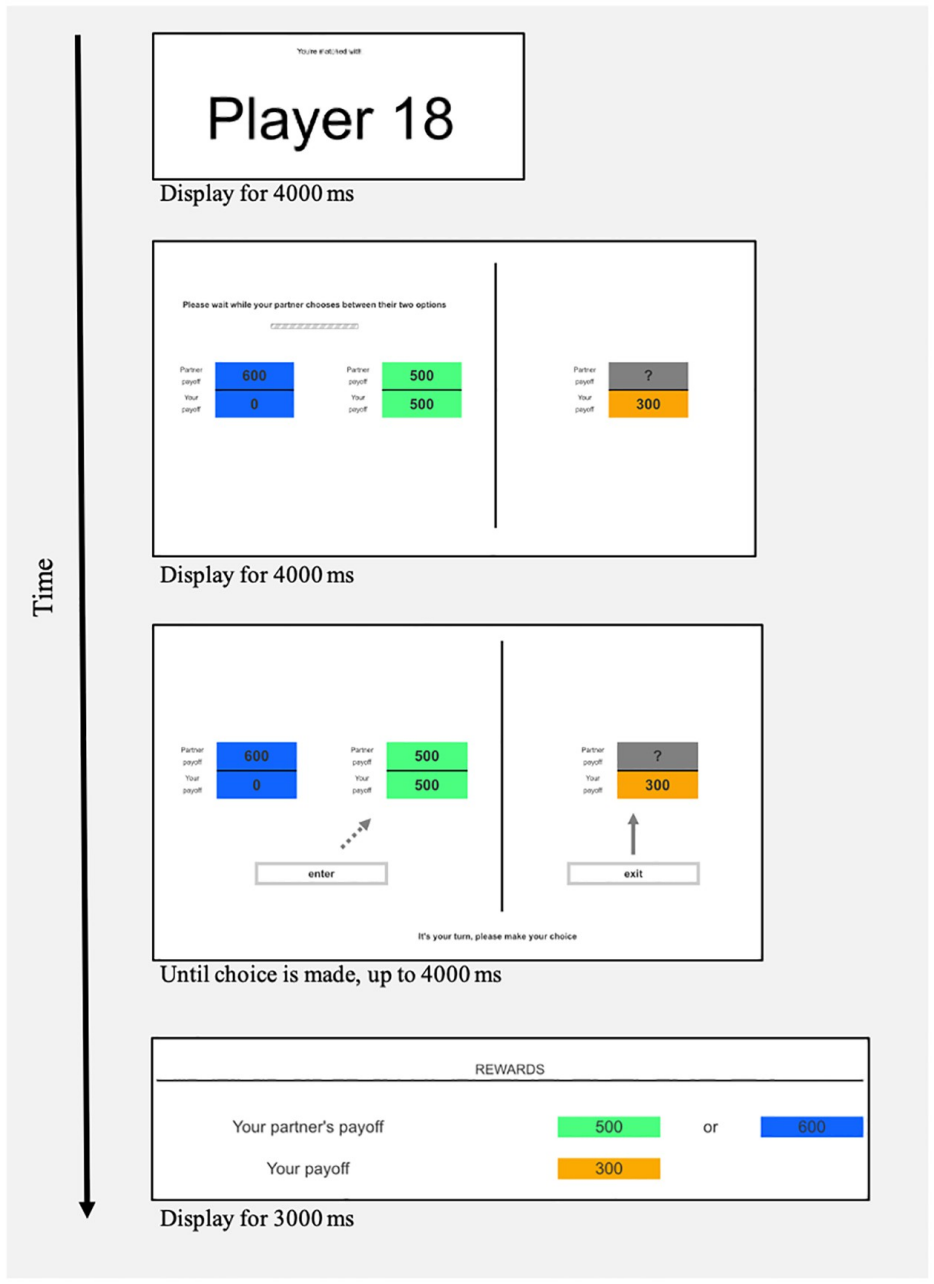
Experiment 2 trial structure. At the beginning of each trial an image of the partner’s player number was displayed, which was either the same (Fixed Partner Condition) or different (Variable Partners Condition) on every trial. Next, two boxes (coloured in green and blue respectively) containing payoff options appeared on the screen, and the participant waited for a fixed duration of 4000 ms while the partner ostensibly selected one of these two boxes. Crucially, participants were led to believe that one of the options presented to the partner was a more or less tempting alternative option. Participants then chose whether to trust (i.e. to select the blue or green box) if they expected that their partner had previously chosen this same option, or to exit (i.e. to select the alternative option, in orange) if they did not trust their partner to have chosen the mutually beneficial option. Participants’ alternative options always entailed a lower reward.

**Fig 6:**
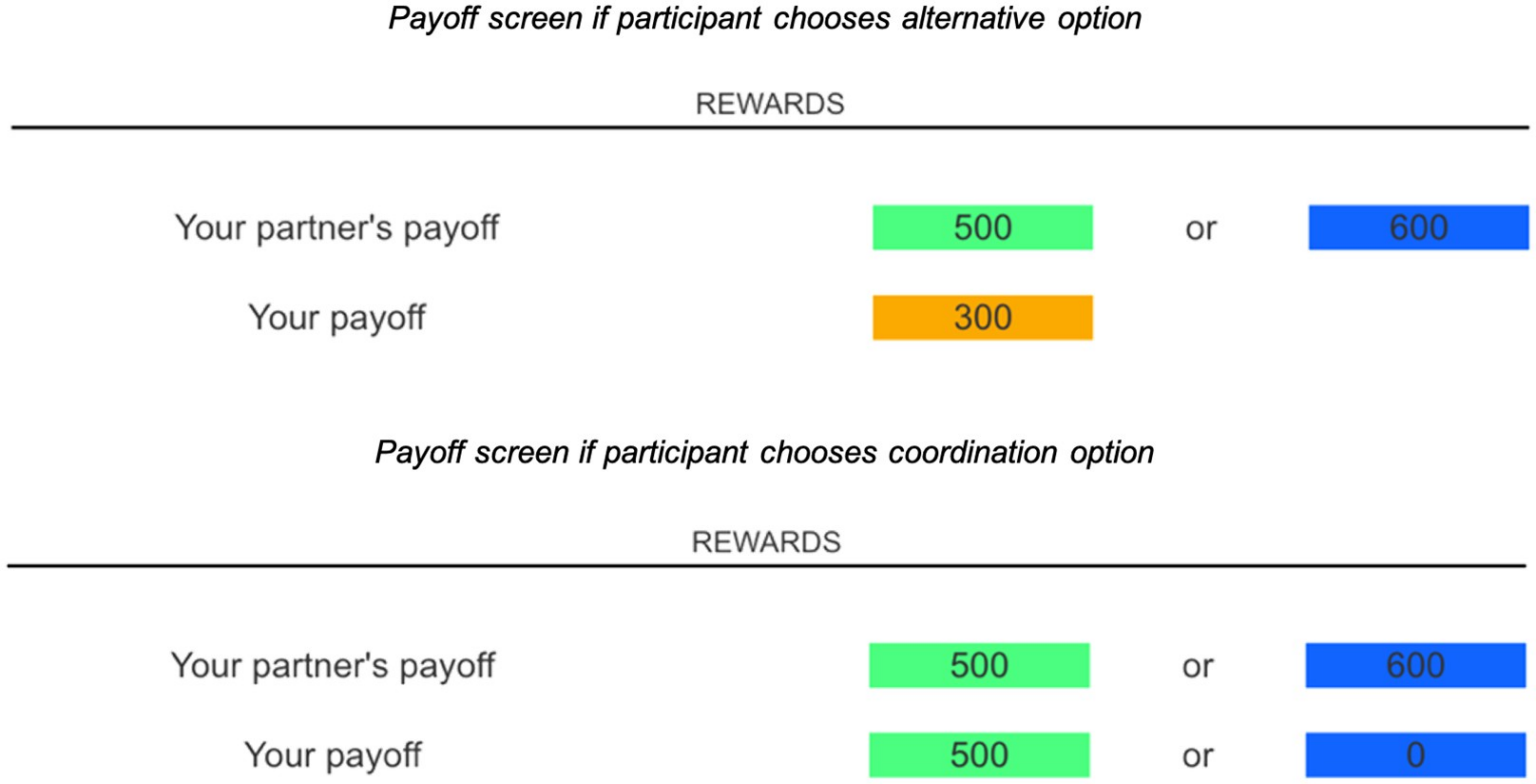
Experiment 2 payoff structure. If the participant chose the alternative option (orange), then payoffs were determined as in the top half of the figure above: the participant received the guaranteed amount (300), and the partner received the amount corresponding to the option s/he had chosen: 600 if s/he had chosen the alternative option (blue) or 500 if she had chosen the coordination option (green). Conversely, if the participant chose the coordination option, the payoffs were determined as in the bottom half of the figure above: If the partner had chosen the coordination option (green), then each player received the corresponding amount (500); if the partner had chosen the then both chosen the alternative option (blue), the partner received the corresponding amount and the participant received 0. Participants should thus only choose the coordination option if they believed their partner had likewise done so. While payoffs for the coordination option and for participants’ alternative options were fixed, we varied the amount associated with the partner’s alternative option unpredictably across trials. Payoffs were displayed for a set amount of time before participants proceeded to the next trial.

The procedure of Experiment 2 was designed visually and procedurally to be as similar as possible to Experiment 1. In particular, the presentation of the partner’s player number showed either the same number (Fixed Partner Condition) or different numbers (Variable Partners Condition) on each trial, depending on the experiment block. Again, participants were instructed that both their own and their partner’s monetary payments at the end of the experiment would depend on the points accumulated during their task, which would vary between £5 and £10 and take maximum 1 hour. The procedure was therefore the same as in Experiment 1, except for the following changes (See Figs 5 and 6). First, after displaying one’s partner’s number, two boxes (colours green and blue) containing payoff options appeared horizontally on the screen (purportedly visible to both participant and partner), one box positioned in the middle of the screen (coordination option) and the other on either the right- or left-hand side of the screen (partner’s alternative option). Each box contained two values representing points: a value on top for the partner’s points and a value on the bottom for the participant’s points. The box in the middle always contained a value of 500 for both partner and participant. The partner’s alternative box always contained some positive value, ‘A’, for the partner and a value of zero for the participant. Value ‘A’ ranged, unpredictably, between 400–700 points in increments of 50. Thus, the differential between the partner’s value in the middle box (500) and the side-aligned box (value A) ranged from 100 points lower to 250 higher. Participants waited while their partner ostensibly selected b7etween one of these two green and blue boxes. The duration of the partner’s selection phase was always 4000 ms, matching the amount of time available to the participant to make their own choice.

Then, an additional box of a different colour (orange) appeared on the empty side of the screen (participant alternative option). The partner’s value in this box was unknown while the participant’s value was either 200 or 400. Thus, participant’s alternative option value was always lower than the central coordination option value. Two buttons simultaneously also appeared below the boxes, one to the left and one to the right-hand side of the screen. One contained the text “Enter” and the other “Exit”; participants clicked the former to choose coordination option and the latter if they wanted to select the guaranteed alternative option.

Participants thus used these buttons to make their choices, based on how they believed their partner had previously behaved, with payoffs for each trial determined as follows. If both participant and partner chose their alternative options, each received the corresponding amount for certain. Conversely, if both chose the coordination option, each received the amount corresponding to coordination. However, crucially, if the participant chose the coordination option and the partner did not, then the partner received the amount corresponding to their tempting alternative option, while the participant received nothing. If the partner chose the coordination option but the participant did not, the partner would nevertheless receive the amount corresponding to the coordination option, and the participant would receive the (lower) guaranteed amount of their alternative option.

Participants therefore had the option of joining their partner (coordination option) if they trusted that their partner had previously chosen this option; or they could exit (alternative option) if they did not trust that their partner had chosen the mutually beneficial option. Their exit option entailed a guaranteed, yet lower, reward. Participants would thus only choose the coordination option if they believed that their partner had also done so. Note that their partners’ rewards were unaffected by the participant’s decisions. While the payoffs for the coordination options and for participants’ alternative options were fixed (as described above), we varied the amount associated with partner’s alternative option unpredictably across trials. This allowed us to measure participants’ trust in their partner at varying levels of reward temptation for their partner; e.g. in some trials, the partner faced a high temptation to not coordinate while in other trials the reward for coordination and non-coordination were identical.

Each trial ended when the participant clicked on one of the buttons to make a choice, or when 4000 ms has elapsed (i.e. they automatically progressed to the next trial, receiving a bonus of zero for the missed attempt). After the participant made their selection, there was a 500 ms delay after which possible payoffs were displayed for 3000ms. The reward display showed the following: the participant’s choice, their partner’s ostensible choice and the rewards for each player.

The experiment lasted approximately 40 minutes, during which participants performed two experimental blocks, one in each condition, in counterbalanced order. In each experimental block, participants first underwent an induction phase consisting of 10 trials: 4 trials for which the alternative option was 100 less than the value for the coordination options; 4 for which it was 50 less; and 2 for which it was the same. There was no perceptible gap between the induction and test phases. As in Experiment 1, in the test phase of each experimental block, there were 64 test trials (80 including those in the induction phase), giving a total 124 trials across the two blocks. Within each block, for each of the 8 different levels of temptation there were 8 trials, drawn in random order. The position of the two options (coordination option and alternative option) varied equally between the left- and right-hand sides of the screen, and the colour of the coordination option varied equally between blue and green.

Participants received no information about their partners’ choices, and were told that their partners would receive no feedback on the choices the participant made until one trial was randomly selected for payment at the end of the experiment. Payment was automatically calculated based on the choice made in the selected trial and participants were paid at the end of the experiment after all participants finished.

## Results

**Fig 7:**
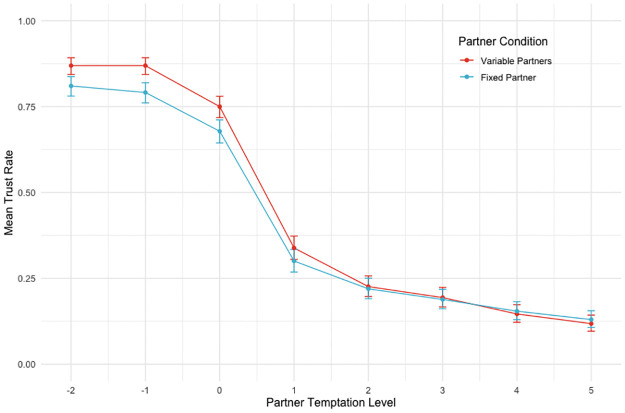
Experiment 2 data. Graphs show mean trust rates (proportion of trials in which participants chose the coordination option) by partner condition for the corresponding temptation level of partners’ alternative option. Points represent where data was collected, and error bars represent 95% confidence intervals calculated using binomial tests. Regression results (see Table 2) show that trust rates were significantly lower when participants coordinated with the same partner (Fixed Partner) on every trial than when coordinating with different partners (Variable Partners) on each trial. In addition, trust rates are significantly decreasing with increases in the level of partners’ temptation of the alternative option.

Table 3 shows the results of two regression models, each including, as in Experiment 1, the respective IV of interest’s corresponding by-subject random coefficient. Fig 7 presents the data from Experiment 2, showing mean trust rates by partner temptation level and by partner condition.

**Table 3:** Analysis results from Experiment 2 using mixed-effects logistic regressions of partner condition and partner temptation level on participants’ choices. Regression models are identical to those used in Experiment 1. Again, Model 1 and Model 2 are used for inferring the effects of partner condition and temptation level, respectively, on trust rates, given the inclusion of their corresponding by-subject random coefficients.

Dependent Variable = Trust Choice	Model 1					Model 2				
			95% CI for odds ratio					95% CI for odds ratio		
	B (SE)	*p* =	Lower	Odds Ratio	Upper	B (SE)	*p* =	Lower	Odds Ratio	Upper
Partner Condition	-0.337	.000	0.589	0.714	0.860	-0.493	.000	0.508	0.611	0.725
	(0.096)					(0.089)				
Partner Temptation Level	-0.927	.000	0.382	0.396	0.409	-2.030	.000	0.082	0.131	0.198
	(0.017)					(0.215)				
Constant	-0.958	.052	1.678	2.607	4.064	1.240	.000	2.239	3.457	5.405
	(0.223)					(0.221)				
By-subject random coefficient	Partner Condition					Partner Temptation Level				
Observations	12,339					12,339				
Log Likelihood	-4,635.10					-3,724.10				
Akaike Inf. Crit.	9,284.20					7,462.30				

Dependent variable is a choice dummy equal to 1 if subject chose trusting option and 0 if alternative option. Partner condition dummy equal to 1 in Fixed Partner Condition and 0 in Variable Partners Condition. Both regressions include as covariates a by-subject random intercept and a by-subject random coefficient for trial number.

Hypothesis 2 predicts higher trust rates in the Fixed Partner Condition than in the Variable Partners Condition. The results of mixed-effects logistic regressions do not corroborate this prediction. In fact, from Model 1, predicted trust rates were significantly lower (*b* = -0.337, *p* = .000) when participants coordinated with the same partner across trials than when coordinating with different partners on every trial. The odds of choosing the coordination option change by 0.714 (95% conf. int.: 0.589, 0.860) when subjects coordinate with a Fixed Partner relative to Variable Partners. This finding is difficult to reconcile with the hypothesis that repeated coordination enhances trust.

As in Experiment 1, we performed a manipulation check by testing the prediction that there would be a negative main effect of partner temptation level. This was because participants should be less likely to expect their partner to coordinate when their partner’s alternative option was more tempting. The results in Model 2 confirm this prediction: at higher partner temptation levels, participants were significantly less likely (*b* = -2.03, *p* = .000) to trust their partner had chosen the coordination option.

Finally, we again ran an additional exploratory statistical test (not shown), with temptation level categorised into two factors and checking for an interaction with partner condition. We found significant main effects for both temptation level and partner condition and a significant interaction between the two. This suggests that participants were (surprisingly) more likely to act as if they expected their partner to defect when facing a fixed versus variable partner, but only in situations in which there was no temptation for their partner to do so.

## Discussion

**Fig 8:**
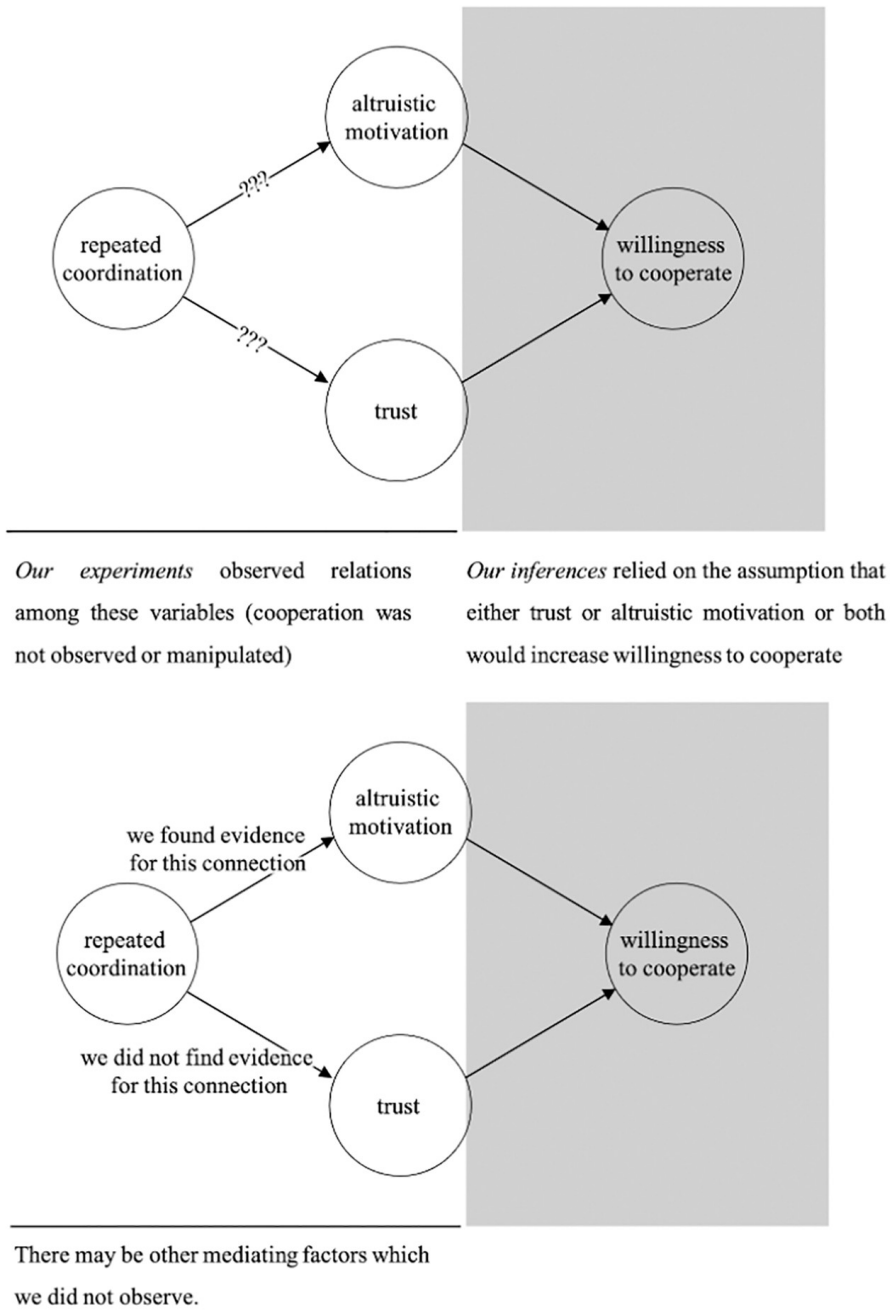
Our research investigated two competing hypotheses for why repeated coordination may boost an agent’s willingness to cooperate with their partner. Our studies relied on the background assumption that either trust or altruistic motivation, or both, would increase agents’ willingness to cooperate with their partners. In each of our two separate studies, we tested a distinct hypothesis and found evidence that repeated coordination boosts altruistic motivation towards one’s partner but found no evidence of an effect of repeated coordination on trust.

The findings from two experiments presented here build upon previous research showing that repeated coordination over time with the same partner can increase people’s willingness to cooperate with that partner–i.e. to coordinate even when doing so is not in their own short-term interest [10, 11, 14]–relative to their willingness to cooperate with changing partners. Our findings (see Fig 8) extend this previous research by illuminating the underlying cognitive and motivational mechanisms underpinning the effects of coordination *per se* upon the willingness to cooperate. Specifically, the results from Experiment 1 provide evidence that pure repeated coordination with the same partner increases altruistic motivation towards that partner (the altruism hypothesis); the results of Experiment 2 do not support the hypothesis that pure repeated coordination (without the history of coordination success) with the same partner increases trust (the trust hypothesis).

Our design also permits us to exclude the possibility that the effect of repeated coordination upon altruism was driven by adherence to specific conventions arising during the experiment, as in one earlier study [11]. This is because the values, colours and positions of the choices varied stochastically from one trial to the next, such that the coordination option could not take on the character of a convention.

The absence of any positive effect of repeated coordination upon trust is consistent with rational decision-making insofar as a partner’s willingness to coordinate when it is in her interest does not directly provide evidence that she would resist tempting alternative offers. The finding that participants in fact exhibited less trust when playing with the same partner on every trial than when playing with a different partner in non-tempting trials is, on the face of it, more surprising. However, it is worth emphasizing that trust requires an element of risk due to dependence on another person. In Experiment 2 the balance of power was against participants–their partner could influence their payoff but they could not influence their partner’s payoff. Given this setup, a participant with variable partners likely expected that their partners will simply follow their own interests and predominantly choose the best options for them. However, in a condition with fixed partner the asymmetry of power might have also led a participant to believe that their partner might want to not only maximize their own payoff, but also to gain competitive advantage over the participant by choosing non-cooperative options even in non-tempting trials. As such our results suggest that in the absence of consistent feedback, repeated interaction when there is a power asymmetry might in fact lead to the decrease rather than increase of trust. The need to explore this and other possibilities provides an important avenue for further research.

Further research on the cognitive and motivational foundations of human cooperativity may also draw upon the novel experimental designs developed here. One key innovation is the technique, employed in both experiments, of seamlessly alternating between coordination problems with aligned interests and social dilemmas without having to change the task structure. Moreover, the task designed for Experiment 2 constitutes an important innovation insofar as it precisely isolates trust as a factor in decision-making, ruling out any influence of altruistic motivations or of expectations of reciprocity that may be confounded with trust in standard economic trust games [30]. This innovation does however also imply a limitation with respect to the possibility of comparing our two studies. This is that the decision to coordinate is up to participants in Experiment 1 but not in Experiment 2. In Experiment 2, participants cannot know whether their decision leads to coordination. Though we designed our tasks to be as similar as possible, our first priority was to separately isolate altruism (Experiment 1) and trust (Experiment 2), leading to an inherent imbalance of decision-making power between the two contexts.

It is also worth reiterating that our experimental procedures did not involve feedback–i.e., participants in Experiment 1 were informed that their partners would receive not feedback about their decisions, and participants in Experiment 2 did not receive feedback about their partners’ decisions. The rationale for this implementation was that the inclusion of feedback would introduce other potential processes which may boost cooperation over and above the processes picked out by the hypotheses we aimed to test. And indeed some studies have shown that feedback and reputational concerns play an important role in sustaining cooperation between participants, perhaps by allowing inferences of trustworthiness and providing incentives to act in others’ interests. The fact that we found no evidence of a direct effect of coordination on trusting behaviour in the absence of feedback suggests that feedback about others’ trustworthiness may play an important role in leading people to draw such inferences. However, the fact that we found a direct effect on participants’ own willingness to bear personal costs suggests that an effect of coordination upon altruistic motivation may play an important role even without feedback in dynamically supporting cooperation in contexts involving imperfectly aligned interests. A fruitful direction for future research would be to manipulate the presence or absence of feedback using our experimental designs.

An additional avenue for future investigation would be to disentangle various more specific hypotheses that could account for agents’ motivation to bear a cost to benefit their partner, as observed in Experiment 1. For example, participants may have other-regarding preferences over their partner’s outcomes, or they may be unwilling to incur obligations to their partner. Insofar as Experiment 1 was designed to test the more general hypothesis that coordination boosts altruistic motivation, the results do not allow us to adjudicate among these and other more specific hypotheses which would generate the same prediction regarding the effect of coordination on cooperation in the context of our Experiment 1. It bears emphasizing that our starting point for the current research was the observation that cooperation in a prisoner’s dilemma can be boosted by increasing trust, by increasing altruism (a willingness to bear a cost for your interaction partner), or by increasing both. Differentiating among additional diverse hypotheses that explain our positive finding in Experiment 1 would be an important next step. A related limitation concerns the extent to which participants believed they might interact with certain partners again in the future, despite this not being indicated in the study design and participant numbers being anonymous. If such beliefs differ when one’s partner is fixed versus variable, controlling for these beliefs in future research might be useful.

In sum, our results support the hypothesis that repeatedly coordinating with a partner increases altruistic motivation towards one’s partner, whereas they do not support the hypothesis that repeated coordination boosts trust. These findings, together with the paradigms introduced in the two experiments, provide new insights and directions for further investigation into the cognitive and motivational underpinnings of human cooperation.

## Supporting information

S1 File Analyses of interaction between temptation to defect and partner condition. (DOCX)
