# Supplementary material for: Coordinated decision-making boosts altruistic motivation—But not trust
Source: PLoS One. 2022 Oct 4;17(10):e0272453. doi: 10.1371/journal.pone.0272453 (PMC9531816; doi:10.1371/journal.pone.0272453)
Supplement: S1 File — (DOCX) [file pone.0272453.s001.docx]

SUPPLEMENTARY MATERIALS S1:
ANALYSES OF INTERACTION BETWEEN TEMPTATION TO DEFECT AND PARTNER CONDITION

**RESULTS**

To investigate the interaction between temptation to defect (by the participant in Experiment 1, or by the interaction partner in Experiment 2) and participant condition (the same partner vs. changing partners) in both Experiments we conducted two additional exploratory mixed logistic regression analyses. For these analyses we transformed the continuous variable reflecting the temptation level into a binary variable taking the value of 0 when there was no temptation to defect, and 1 when there was a temptation to defect. Specifically, we assumed that there is no temptation to defect if choosing a collaborative option leads to higher or equal reward as defecting, and that there is a temptation if defecting leads to higher reward than cooperating.

The models included Partner Condition (0: always the same vs. 1: always different), Temptation Class (0: No temptation vs. 1: Temptation) and their interaction as fixed factors. We included a random effect to allow intercept to vary by participant.

**Table S1**. Analysis results from each experiment using mixed-effects logistic regressions of partner condition and an indicator of positive temptation level (0= no temptation to defect present; 1= temptation to defect present) including their interaction. Regression models are identical to those used in each experiment, barring this modification of the temptation level variable.

| **Dependent variable = Altruism choice** | **Experiment 1 (altruism)** | | | | | **Experiment 2 (trust)** | | | | |
| --- | --- | --- | --- | --- | --- | --- | --- | --- | --- | --- |
|  |  |  | 95% CI for odds ratio | | |  |  | 95% CI for odds ratio | | |
|  | B (SE) | *p* = | Lower | Odds Ratio | Upper | B (SE) | *p* = | Lower | Odds Ratio | Upper |
|  |  |  |  |  |  |  |  |  |  |  |
| Partner Condition | 0.027 | .839 | 0.75 | 0.97 | 1.27 | -0.597 | .000 | 0.464 | 0.55 | 0.65 |
|  | (0.135) |  |  |  |  | (0.088) |  |  |  |  |
| Temptation Class | -3.988 | .000 | 0.022 | 0.027 | 0.035 | -4.162 | .000 | 0.013 | 0.016 | 0.019 |
|  | (0.122) |  |  |  |  | (0.092) |  |  |  |  |
| Temptation Class * Partner Condition | -0.390 | .012 | 0.50 | 0.68 | 0.92 | 0.546 | .000 | 1.39 | 1.73 | 2.15 |
|  | (0.155) |  |  |  |  | (0.111) |  |  |  |  |
| Constant | 3.531 | .000 | 18.62 | 35.12 | 66.24 | 2.261 | .000 | 6.44 | 9.60 | 14.29 |
|  | (0.324) |  |  |  |  | (0.203) |  |  |  |  |
|  |  |  |  |  |  |  |  |  |  |  |
| By-subject random coefficient | None | | | | | None | | | | |
| Observations | 8,320 | | | | | 12,339 | | | | |
| Log Likelihood | -2994.67 | | | | | -4625.59 | | | | |
| AIC | 5999.35 | | | | | 9261.18 | | | | |
| Dependent variable is a choice dummy equal to 1 if subject chose altruistic option and 0 if alternative option. Independent variables are: (1) Partner Condition dummy equal to 0 in Variable Partners Condition and 1 in Fixed Partner Condition, (2) Temptation Class dummy equal to 0 in No Temptation Condition, and 1 in Temptation Condition. Both regressions include as covariates a by-subject random intercept. The analyses were conducted in R with lme4 package. | | | | | | | | | | |
|  |  |  |  |  |  |  |  |  |  |  |

**DISCUSSION**

Our exploratory results confirmed that there was a significant interaction between Partner Condition and Temptation Class in both experiments. In Experiment 1 the interaction reflected that participants were more likely to cooperate with a stable partner than with changing partners but only when there was a temptation to defect. On the other hand, in Experiment 2 participants were (surprisingly) more likely to defect when facing a stable partner than a changing partner, but only in a situation in which there was no monetary incentive to do so (no temptation).
